# Development of a Cigarette Smoking Obscenity Scale (CSOS) in adolescents: an exploratory sequential mixed method design

**DOI:** 10.34172/hpp.2020.21

**Published:** 2020-03-30

**Authors:** Khadijeh Keshavarzian, Haidar Nadrian, Asghar Mohammadpoorasl

**Affiliations:** ^1^Department of Statistics and Epidemiology, Tabriz University of Medical Sciences, Tabriz, Iran; ^2^Department of Health Education and Promotion, Tabriz University of Medical Sciences, Tabriz, Iran; ^3^Social Determinants of Health Research Center, Tabriz University of Medical Sciences, Tabriz, Iran; ^4^Research Center of Psychiatry and Behavioral Sciences, Tabriz University of Medical Sciences, Tabriz, Iran

**Keywords:** Instrumentation, Adolescent behavior, Obscenity, Cigarette smoking

## Abstract

**Background:** Considering the increasing prevalence rate of smoking among Iranian adolescents,and recent evidence on the relationship between obscenity of smoking and tendency of adolescents towards the behavior, there is a need for an appropriate measurement tool to measure the level of obscenity on cigarette smoking among adolescents. This study was conducted to develop a valid and reliable questionnaire for measuring the obscenity of cigarette smoking in adolescents.

**Methods:** This study was conducted in Tabriz, Iran, using an exploratory sequential mixed methods design. To explain the concept of obscenity and develop the questionnaire’s items, 18 students attended semi-structured individual interviews and 13 others took part in focus group discussions (FGDs) in three groups of 4-5. Extracting and summarizing the codes derived from the interviews, an item pool was developed, from which the initial draft of the scale was provided.Next, the psychometric properties of the scale were assessed using face, content, construct, and predictive validity, as well as internal consistency, and reliability in a sample of 1013 high school students.

**Results:** The 22-item Cigarette Smoking Obscenity Scale (CSOS) was developed based on thecodes derived from qualitative data. Explanatory factor analysis revealed five-factor structure(Negative Attitude; Negative Consequence; Negative Valuation; Inappropriate Relationship; Agateway to addiction). In confirmatory factor analysis, the χ^2^/df ratio was 3.911 for the CSOS five-factor structure. Suitable values were obtained for the goodness of fit indices (GFI = 0.88,AGFI = 0.85, NFI = 0.87, IFI = 0.90, CFI = 0.90, RFI = 0.85, and RMSEA = 0.072). The Cronbach’s alpha and intraclass correlation (ICC) coefficients for the constructs ranged between 0.77 to 0.90 and 0.80 to 0.91, respectively.

**Conclusion:** The validity and reliability of the CSOS was appropriate; therefore, it can be used infuture studies as a suitable tool for measuring the obscenity of cigarette smoking in adolescents.

## Introduction

The use of tobacco (particularly cigarette) leads to several preventable deaths worldwide.^1^ The number of smoking-related deaths is estimated to rise from 4.8 million in 2006 to 8.3 million by 2030.^2^ The World Health Organization (WHO) reports indicate a shift in geography of smoking from the developed to developing countries, which is more acute in Asian countries.^3^

The prevalence rate of adolescent smoking is reported to be 8% in India,^4^ 15.17% in Saudi Arabia,^5^ and 29.6% (32.6% in boys and 26.7% in girls) in Greece.^6^ This rate among adolescents is reported between 2.5% and 17% in various studies conducted in Iran.^7,8^ This broad range is due to different definitions of “smokers,” enrollment of participants at different age groups, and selection of different study locations. Despite these differences, one can easily observe the increasing prevalence of smoking among Iranian adolescents.^9^ Also, the studies of Ayatollahi et al^10^ and Mohammadpoorasl et al^11,12^ have shown the high rate of smoking among Iranian adolescents.

The word “obscenity” means indecency and depraving. An act is considered to be obscene if it is deemed indecent and unpleasant from an individual point of view. Obscenity can be considered a very effective factor in preventing inclination towards smoking tobacco (cigarettes). There is no globally accepted definition for obscenity. It is a subjective concept and its definition highly depends on the dominant cultures in different societies; hence, different definitions have been provided for obscenity in different cultures.^13^

Khayyati et al argued that higher prevalence of smoking among boys (than in girls) in Iran may be due to their higher freedom, as well as higher obscenity of this behavior in girls.^14^ Yousefi et al conducted a two-phase study in Bushehr Province, and found that decrease in the social obscenity of tobacco use in women is one of the main causes of changes occurred in the smoking habits of women.^15^ Mohammadkhani et al found that negative attitude towards smoking and the obscenity of the behavior from a social point of view may reduce the likelihood of smoking.^16^ In addition, Mohammadpoorasl et al^17^ stated that obscenity and social acceptance can significantly slow down progress towards advanced stages of smoking.

Although the association of obscenity of cigarette smoking and smoking behavior is emphasized in the above-mentioned studies, to the best of our knowledge, there is still no tool to measure the obscenity of smoking in any society.

So, considering the increasing prevalence rate of smoking among Iranian adolescents, there is a need for an appropriate measurement tool to measure the level of obscenity on cigarette smoking among adolescents. This study was conducted to develop a valid and reliable questionnaire for measuring the obscenity of cigarette smoking in adolescents.

## Materials and Methods

This study was conducted between February 2017 and March 2018 in Tabriz, Iran, using an exploratory sequential mixed methods design. At first, the qualitative data were collected and the codes were extracted from the interviews, and then the quantitative data were collected. Eventually, all data were integrated at the interpretation (discussion and conclusion section of the study) phase of the study. The study population consisted of all female and male high school students in Tabriz. The researchers visited selected schools located at the fourth educational district of Tabriz, explained the research objectives to the selected students, and obtained the students’ informed consent to participate in the study.

### A. Qualitative part

*Participants and sampling*

Focus group discussions (FGDs) and semi-structured individual interviews were conducted with the participants on the obscenity of cigarette smoking, and the interviews were recorded with their permission. The main question initially asked from the participants was ‘how would you explain cigarette smoking?’, and then some probing questions were asked according to the interview schedule and the participants’ answers. The participants were selected using purposive sampling, and the sampling process continued until data saturation.

*Data collection and analysis*

A total of 18 students attended the individual interviews and 13 others took part in FGDs in three groups of 4-5. Each interview lasted for 35-55 minutes, while each FGD took about 35-40 minutes. The researchers played the recorded interviews and transcribed them word-by-word in a Microsoft Word format. The data were then inserted into MAXQDA-10 and the codes were extracted. Further details on the methodology of this phase are published elsewhere.^18^

As a complement for the qualitative part and in order to finding out a richer item pool, we also referred to six conveniently selected classes in two schools (a male, and a female school, consisting of 148 students) and asked the students to complete the following sentence using at least 3 words. We wrote down the sentence “cigarette smoking is … for me” on the classes’ boards. Their answers (i.e., sentences written on the boards) (120 sentences) were summarized and analyzed, and with the codes extracted (739 codes) from the interviews, constituted an item pool to design the initial draft of the scale (859 codes). In several sessions, the team of research examined the items, which were finally reduced by 28 items. These remaining items were reviewed in a final session to diminish the items in terms of number. Eventually, with considerations on relevancy, appropriateness, and the cases of redundancies, we reduced the number of items to 26. A response format based on a five-point Likert-type scaling (“completely agree”, “agree”, “no idea”, “disagree”, and “completely disagree”) was designed.

### B. Quantitative part

In this part, the psychometric properties of the Cigarette Smoking Obscenity Scale (CSOS) were assessed using face, content, construct, and predictive validity, as well as internal consistency, and reliability.

*Content validity*

The face and content validity of the CSOS were assessed using expert opinions with a qualitative method. The questionnaire was provided to 22 health education and promotion experts, psychiatrists, psychologists, and epidemiologists who had experiences on smoking research. These experts were asked to assess the scale in terms of appropriate words use, proper placement of items, correct use of grammatical structures, and the compliance of the test with the measured concept (face validity). Thirteen (out of 22) experts responded to the invitation. Based on the experts’ responses, four items were deleted, and thus, the number of items dropped down from 26 to 22.

*Construct validity*

*Exploratory factor analysis*

Construct validity was assessed in this phase. Exploratory factor analysis (EFA) was used to see whether the logical structures defined for the CSOS could be derived from the prepared questions and items. The Bartlett’s test of sphericity and Kaiser-Meyer-Olkin (KMO) test were used to the measure of sampling adequacy (KMO values >0.7 indicate a desirable adequacy of the data, and *P* values <0.05 in Bartlett’s test confirm this adequacy). A cut-off point of 0.3^19^ was determined as the minimum factor loading value. In addition, 5 factors were considered for the scale, and a scree-plot was used in EFA. To perform the EFA, the data derived from 450 questionnaires were used and principal components analysis with Varimax rotation was carried out in IBM SPSS Statistics 24.0 (IBM SPSS Statistics, Inc., Armonk, USA).

*Confirmatory factor analysis*

The confirmatory factor analysis (CFA) model was developed based on the data collected from 563 students. CFA with maximum likelihood was performed in Amos 24 (IBM^®^ SPSS^®^ Amos™ 24) to examine the 5-factor structure of the scale, and goodness of fit indices including root mean square error approximation (RMSEA), goodness of fit index (GFI), normed fit index (NFI), relative fit index (RFI), and incremental fit index (IFI).

*Reliability*

In addition, to examine reliability, the CSOS was completed by 60 students. Internal consistency and stability were used to assess the overall reliability of the scale. Internal consistency was assessed by calculating Cronbach’s alpha coefficients. Moreover, the test-retest method was used to assess the reliability of the scale. To this end, the scale was completed within a two-week interval, and then the scores obtained in these two stages were compared using the intra-class correlation (ICC) coefficients.

*Predictive validity*

Finally, the predictive validity of the scale was conducted to assess the relationships between obscenity and smoking behavior status in 1013 students. A multi-stage cluster sampling was used to select the participants. At first, 8 high schools were randomly selected from all the high schools in Tabriz, considering the type of school and the gender of students. Then, with regard to the number of students in each school and field of study, 28 classes were selected as clusters and all students in the selected classes were included in the study. To determine the predictive validity of the scale, the variables of inclination towards smoking, age, gender, socioeconomic status, having a smoker friend, and having a smoker in the family were considered as potential confounding variables.

## Results

Based on the qualitative analysis results, 739 codes and 120 statements were obtained.

Regarding the face and content validity of the CSOS, the experts offered their corrective opinions, and using these comments and opinions, the researchers omitted duplicate questions, added necessary questions, and changed the format of some questions. Finally, the 22-item CSOS was prepared, and its face and content validity were confirmed. It should be noted that no major change was made to the scale with regard to the feedbacks received from the students.

To perform EFA, the data derived from 450 participants were used. The KMO value was 0.93 and Bartlett’s p-value was smaller than 0.001; therefore, the data were suitable for conducting factor analysis.

**Figure 1:**
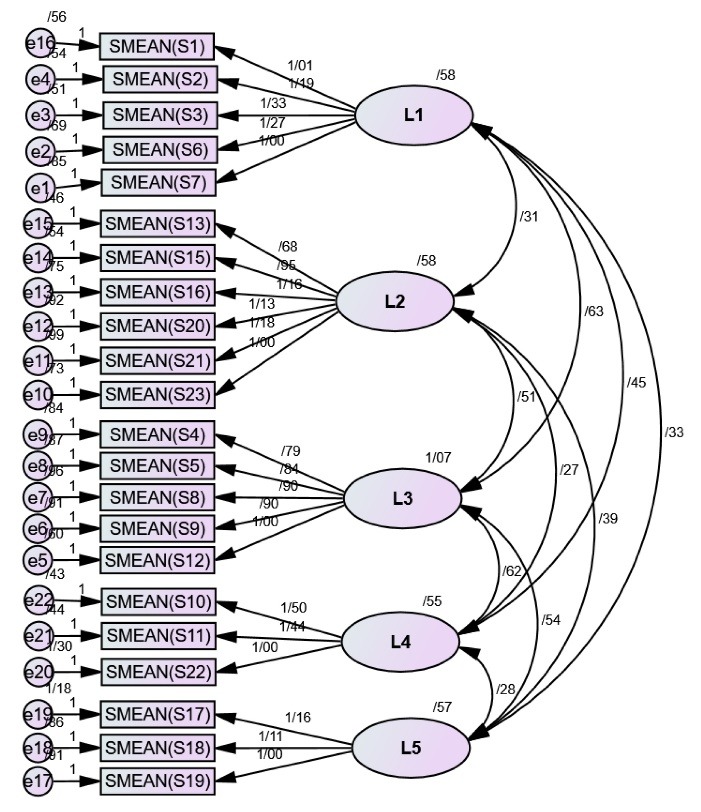
Five-factor structural model of the Cigarette Smoking Obscenity Scale. L1: Negative Attitude; L2: Negative Consequence; L3: Negative Valuation; L4: Inappropriate Relationship; L5: A gateway to addiction.

The 5 factors related to the scale included “negative attitude towards smoking and smokers” (5 items), “negative consequences of smoking” (6 items), “negative value placed on smokers by society” (5 items), “inappropriate relationship of smokers with others” (3 items), and “smoking; a gateway to addiction” (3 items). The item “smoking does not harm one’s physical appearance” was omitted due to its low explained variance. The total explained variance for the designed questionnaire was 63.99 (Table 1). Then, a CFA was conducted on 563 students. As shown in Table 2, the χ^2^/*df* ratio was smaller than 5; hence, the research model fits the data well. In addition, the goodness of fit indices of NNFI, NFI, AGFI, GFI, IFI, RFI, CFI, and RMSEA confirmed the goodness of the model. Therefore, the final model can be used to confirm the questionnaire’s factors (Figure 1). It should be noted that the CFA coefficients were significant for all constructs of the CSOS (5 factors).

Regarding the reliability of the scale, the Cronbach’s alpha coefficient values for the research constructs ranged between 0.77 and 0.9. Many previous studies^20,21^ have also used Cronbach’s alpha to show the reliability of their scales. The ICC values calculated to assess the stability of the scale are presented in Table 3.

Results for predictive validity of the scale (are presented in Table 4. Higher scores indicate higher obscenity and lower scores indicate lower obscenity of smoking from the students’ point of view. Based on the results, there was a significant relationship between the obtained obscenity values and the students’ smoking behaviors (*P* < 0.001). In other words, inclination towards smoking (smoking behavior) decreases as the obscenity values increase. Therefore, the predictive validity of the CSOS was confirmed.

## Discussion

In this study, an exploratory sequential mixed methods design was used to develop a scale for measuring the obscenity of smoking from the adolescents’ point of view. Exploratory sequential designs are useful for conducting studies on the phenomena that originate from the context of societies, and for designing and testing a tool to identify an unknown phenomenon.^22^ In this study, a 22-item CSOS was designed using the codes extracted from the qualitative data analysis.

The face and content validity of the scale were assessed using a qualitative method. In this regard, the experts offered their corrective opinions, and using these comments and opinions, the researchers omitted duplicate items, added necessary items, and changed the format of some items. The qualitative results of the face and content validity were confirmed. In addition, no significant change was made in the scale with regard to the feedbacks received from the students. The construct validity of the questionnaire was also assessed using exploratory and confirmatory factor analyses.

Based on the EFA results, the 5 factors related to the scale included “negative attitude towards smoking and smokers,” “negative consequences of smoking,” “negative value placed on smokers by society,” “inappropriate relationship of smokers with others,” and “smoking; a gateway to addiction.” The total explained variance for the designed questionnaire was 63.99.

The KMO (0.93) and Bartlett’s test (*P* < 0.001) results confirmed the suitability of the model for conducting factor analysis. Ghasemi et al^23^ (KMO=0.91; Bartlett’s test, *P* < 0.001) and Shahbazi et al^24^ (KMO=0.81; Bartlett’s test, *P* < 0.001) also confirmed the suitability of their models for factor analysis. In addition, a cut-off point of 0.3 was determined as the minimum factor loading value, and the item “smoking does not harm one’s physical appearance” was omitted, because its factor loading was below this value. The CFA results confirmed the goodness of fit of the CSOS in Iranian society. Similar to the present findings, the questionnaire designed by Shahbazi et al^24^ also had an acceptable goodness of fit.

Internal consistency and stability were used to assess the overall reliability of the scale. Internal consistency was assessed by calculating Cronbach’s alpha coefficient. Based on the results, the overall reliability of the designed CSOS was suitable. The Cronbach’s alpha coefficient values for the research constructs ranged between 0.77 and 0.9. This confirms the reliability of the CSOS. Similar to the present findings, the Cronbach’s alpha coefficient values ranged between 0.77 and 0.95 in the study of Ghasemi et al.^23^

The test-retest method was used to assess the stability of the scale. The obtained ICC values ranged between 0.80 and 0.91; therefore, the CSOS had an acceptable stability. This suggests that the scale can probably yield reliable results at different times and places. The predictive validity of the scale was also assessed. Based on the results, there was a significant relationship between the obtained obscenity values and the students’ smoking behaviors. In other words, inclination towards smoking (smoking behavior) decreases as the obscenity values increase. Therefore, the predictive validity of the scale was confirmed, as well. Mohammadpoorasl et al,^17^ introduced obscenity as an important factor affecting adolescents’ inclination towards smoking, and concluded that low obscenity levels may lead to the adoption of a positive attitude towards smoking, and this ultimately increases smoking rates. This instrument is practical as it is developed based on adolescents’ understanding of the concept of obscenity, and the examination of their viewpoints through qualitative research. CSOS also has a desirable reliability and validity, and is easy to be used and understood, and it needs only 10-15 minutes to be completed.

## Conclusion

The model fit indices and the results of reliability reported in the present study may be considered as evidence for validity and reliability of the CSOS. To the best of our knowledge, this is the first scale in the literature to assess the obscenity of cigarette smoking among adolescents. School health practitioners and healthcare providers may use this scale to find valid and reliable data on cigarette smoking obscenity while designing cigarette prevention/cessation programs among adolescents.

## Ethical approval

This research was approved by the Ethics Committee in Tabriz University of Medical Sciences (ethical approval code: IR.TBZMED.REC.1396.1067). The participants were told about the aim of study and were assured on the confidentiality of data. All participants and one of their parents signed a consent form before data collection.

## Competing interests

The authors declare no competing interests.

## Funding

This work received grant from Tabriz University of Medical Sciences (Grant no: 1396. 981/23/1).

## Authors’ contributions

Study design: All authors. Study conduct: HN, KK, and AM. Data collection: KK and AM. Data analysis: AM, KK, and HN. Data interpretation: All authors. Drafting manuscript: AM and KK. Revising manuscript and content: HN, KK, and AM. Approving final version of manuscript: All authors. AM takes responsibility for the integrity of the data analysis.

## Acknowledgments

We thank all students participated in the study.

**Table 1:** Items and the five-factor structure of the Cigarette Smoking Obscenity Scale

**Items**	**Factor 1 (Negative attitude)**	**Factor 2 (Negative consequence)**	**Factor 3 (Negative valuation)**	**Factor 4 (Inappropriate relationship)**	**Factor 5 (A Gateway to addiction)**
1. Smokers are offenders.	0.792				
2. Smokers do not comply with ethical principles.	0.774				
3. Smokers are unaccountable to people around them.	0.731				
4. Smokers do not respect for themselves and others.	0.619				
5. Smokers seem to be stubborn.	0.537				
6. Smoking harms one’s health.		0.671			
7. Smoking is a waste of money.		0.778			
8. Smoking means destroying your future.		0.678			
9. Smoking is absurd.		0.672			
10. A majority of smokers are narrow-minded.		0.439			
11. Smokers are irresponsible for their own health.		0.683			
12. Society does not trust smokers.			0.567		
13. Smokers are stigmatized by community members.			0.724		
14. Smokers lose their good friends.			0.548		
15. People think that smokes come from ignorable families.			0.723		
16- Smoking reduces the value placed by society on a person.			0.681		
17. Smokers do not have good relationships with their family members.				0.785	
18. Smokers cannot adapt with their families.				0.778	
19. Smokers do not respect the privacy of others.				0.519	
20. Smoking is not the beginning of drug use.					0.763
21. Smoking is not something to be embarrassed for.					0.653
22. Smoking is not addictive.					0.647
The proportion of variance explained	39.99	10.48	4.95	4.70	3.87

**Table 2:** Fitness indices to confirm the suitability of the studied pattern

**χ 2**	**df**	***P***	**χ 2/df**	**GFI**	**AGFI**	**RMSEA (95% CI)**	**NFI**	**RFI**	**IFI**	**CFI**
779/664	199	<0.001	3.911	0.88	0.85	0.072 (0.067: 0.077)	0.87	0.85	0.90	0.90

GFI, goodness of fit index; AGFI, adjusted goodness of fit index; RMSEA, root mean square error of approximation; NFI, normed fit index; RFI, relative fit index; CFI, comparative fit index; IFI, incremental fit Index.

**Table 3:** The results of the reliability of the Cigarette Smoking Obscenity Scale

	**No. of questions**	**Range**	**Mean**	**SD**	**Skewness**	**Kurtosis**	**Cronbach' alpha**	**ICC**
Negative attitude	5	20	11.97	4.80	0.456	-0.468	0.90	0.83
Negative consequence	6	24	24.12	5.14	-1.01	-0.669	0.77	0.80
Negative valuation	5	20	15.51	5.12	-0.165	-0.743	0.87	0.82
Inappropriate relationship	3	12	8.09	3.24	0.193	-0.758	0.85	0.91
A gateway to addiction	3	12	10.57	3.02	-0.373	-0.423	0.79	0.85

**Table 4:** Cigarette smoking status and its relationship with Cigarette Smoking Obscenity

**Cigarette smoking status**	**No.**	**%**	**Score of smoking obscenity**	***P*** **value**
Never used	687	69.25	74.33 ± 15.58	<0.001
Only a few packs or 1-2 of cigarettes	130	13.10	66.79 ± 16.70	
More than 2 yarns but less than 100 cigarettes	85	8.57	58.78 ± 15.89	
At least one yarn a month and more than a hundred cigarettes	25	2.52	54.04 ± 15.81	
Every day of the week or on most days of the week	42	4.23	53.12 ± 14.17	
Used to be a smoker, but I quited	23	2.32	62.96 ± 19.62	

## References

[1] Méndez D, Alshanqeety O, Warner KE (2013). The potential impact of smoking control policies on future global smoking trends. Tob Control.

[2] Lim HK, Ghazali SM, Kee CC, Lim KK, Chan YY, Teh HC (2013). Epidemiology of smoking among Malaysian adult males: prevalence and associated factors. BMC Public Health.

[3] Kan MY, Lau M (2010). Minor access control of Hong Kong under the framework convention on tobacco control. Health Policy.

[4] Khubchandani J, Sharma M, Huston D, Tahiliani J (2017). Tobacco use related attitudes and behaviors in Indian Adolescents: association with school-based prevention education. Health Promot Perspect.

[5] Al-Zalabani A, Kasim K (2015). Prevalence and predictors of adolescents’ cigarette smoking in Madinah, Saudi Arabia: a school-based cross-sectional study. BMC Public Health.

[6] Sichletidis LT, Chloros DA, Tsiotsios AI, Spyratos DG (2009). Prevalence and risk factors for initiation of smoking in Greek high-school students. Int J Environ Res Public Health.

[7] Ansari-Moghaddam A, Rakhshani F, Shahraki-Sanavi F, Mohammadi M, Miri-Bonjar M, Bakhshani NM (2016). Prevalence and patterns of tobacco, alcohol, and drug use among Iranian adolescents: a meta-analysis of 58 studies. Child Youth Serv Rev.

[8] Mohammad-Alizadeh-Charandabi S, Mirghafourvand M, Tavananezhad N, Karkhaneh M (2015). Prevalence of cigarette and water pipe smoking and their predictors among Iranian adolescents. Int J Adolesc Med Health.

[9] Nazarzadeh M, Bidel Z, Ayubi E, Bahrami A, Jafari F, Mohammadpoorasl A (2013). Smoking status in Iranian male adolescents: a cross-sectional study and a meta-analysis. Addict Behav.

[10] Ayatollahi SA, Mohammadpoorasl A, Rajaeifard A (2004). Psychological predictors of transition in different stages of cigarette smoking. Journal of Ardabil University of Medical Sciences.

[11] Mohammadpoorasl A, Fakhari A, Rostami F, Shamsipour M, Rashidian H, Goreishizadeh MA (2010). Predictors of transition in different stages of smoking: a longitudinal study. Addict Health.

[12] Mohammadpoorasl A, Nedjat S, Fakhari A, Yazdani K, Rahimi Foroushani A, Fotouhi A (2012). Smoking stages in an Iranian adolescent population. Acta Med Iran.

[13] Ong R (2009). Policing obscenity in Hong Kong. J Int Commer Law Technol.

[14] Khayyati F, Taymoori P, Mohammadpoorasl A, Allahverdipour H, Asghari Jafarabadi M (2016). Underlying predictors of tobacco smoking among Iranian teenagers: generalized structural equation modeling. Int J Pediatr.

[15] Yousefi F, Darabi H, Nabipour I, Assadi M, Vahdat K, Kardeh E (2014). Prevalence of tobacco smoking in Bushehr province: comparison of two phases of the Persian Gulf healthy heart study. Iran South Med J.

[16] Mohammadkhani S, Rezaei Jamaloei H (2016). Relationship between cigarette and hookah smoking with individual, family and social factors in adolescents. Journal of Sabzevar University of Medical Sciences.

[17] Mohammadpoorasl A, Bahari A, Marin S, Hajizadeh M (2019). Obscenity of cigarette and hookah smoking in Iranian adolescents: a longitudinal school-based study. Int J Prev Med.

[18] Keshavarzian K. Developing cigarette and hookah obscenity measurement questionnaire for adolescents: A mixed-method research with a sequential exploratory approach [thesis]. Tabriz: Faculty of Health, Tabriz University of Medical Sciences; 2020. [Persian].

[19] Hadi NU, Abdullah N, Sentosa I (2016). An easy approach to exploratory factor analysis: marketing perspective. J Educ Soc Res.

[20] Yari A, Nadrian H, Rashidian H, Nedjat S, Esmaeilnasab N, Doroudi R (2014). Psychometric properties of the Persian version of Social Capital Questionnaire in Iran. Med J Islam Repub Iran.

[21] Nadrian H, Nedjat S, Taghdisi MH, Shojaeizadeh D (2014). Urban traffic-related determinants of health questionnaire (UTDHQ): an instrument developed for health impact assessments. Med J Islam Repub Iran.

[22] Creswell JW, Plano Clark VL. Designing and Conducting Mixed Methods Research. 2nd ed. Thousand Oaks, CA: Sage Publications; 2011. p. 86-9.

[23] Ghasemi M Sabzmakan L, Asghari Jafarabadi M (2017). Psychometric properties of a protection motivation theory based questionnaire for tobacco use in male adolescents. Payesh.

[24] Shahbazi Sighaldeh S, Baheiraei A, Ebadi A, Khaki I, Kelishadi R, Majdzadeh R (2019). Development and psychometric properties of the Hookah Smoking Initiation for Women Questionnaire (HIWQ). Health Promot Int.

